# Supplementary figures and images for: Label dependency modeling in Multi-Label Naïve Bayes through input space expansion
Source: PeerJ Comput Sci. 2024 Dec 10;10:e2093. doi: 10.7717/peerj-cs.2093 (PMC11784536; doi:10.7717/peerj-cs.2093)

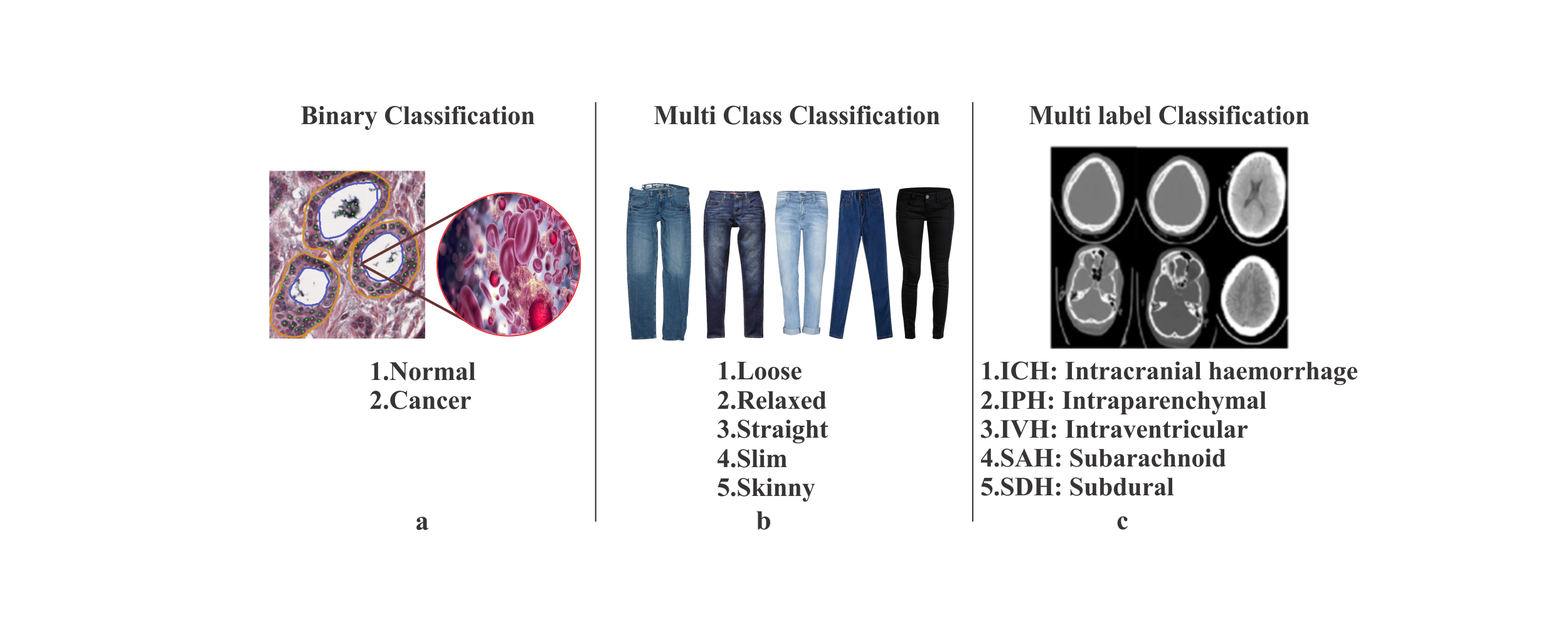

Supplement: Supplemental Information 3 [file peerj-cs-10-2093-s003.png]
